# Surgical perspectives from a prospective, nonrandomized, multicenter study of breast conserving surgery and adjuvant electronic brachytherapy for the treatment of breast cancer

**DOI:** 10.1186/1477-7819-9-30

**Published:** 2011-03-07

**Authors:** William C Dooley, Ozer Algan, Kambiz Dowlatshahi, Darius Francescatti, Elizabeth Tito, J David Beatty, Art G Lerner, Betsy Ballard, Susan K Boolbol

**Affiliations:** 1 University of Oklahoma Health Sciences Center, 825 NE 10th Street Suite 4500, Oklahoma City, OK 73104, USA; 2 Department of Radiation Oncology, University of Oklahoma Health Sciences Center, 825 North East 10th Street Suite 1430, Oklahoma City, OK 73104, USA; 3 Rush University Medical Center, 60 E Delaware Place Suite 1400, Chicago, IL 60611, USA; 4 Rush University Medical Center, 1725 West Harrison Street Suite 810, Chicago, IL 60612, USA; 5 Rhode Island Hospital, Providence, RI 02903, USA; 6 Enterprise Surgical, 91 Washington St Unit 302, Taunton, MA 02780, USA; 7 Swedish Cancer Institute, Comprehensive Breast Center, Swedish Medical Center, 1600 East Jefferson St. Suite 305, Seattle, WA 98122, USA; 8 Dickstein Cancer Center, White Plains Hospital, White Plains, 4 Longview Ave, NY 10601, USA; 9 Holy Cross Medical Center, 2101 Medical Parks Drive Suite 304, Silver Spring, MD 20902, USA; 10 Beth Israel Medical Center, 10 Union Square East, New York City, NY 10003, USA

## Abstract

**Background:**

Accelerated partial breast irradiation (APBI) may be used to deliver radiation to the tumor bed post-lumpectomy in eligible patients with breast cancer. Patient and tumor characteristics as well as the lumpectomy technique can influence patient eligibility for APBI. This report describes a lumpectomy procedure and examines patient, tumor, and surgical characteristics from a prospective, multicenter study of electronic brachytherapy.

**Methods:**

The study enrolled 65 patients of age 45-84 years with ductal carcinoma or ductal carcinoma in situ, and 44 patients, who met the inclusion and exclusion criteria, were treated with APBI using the Axxent^® ^electronic brachytherapy system following lumpectomy. The prescription dose was 34 Gy in 10 fractions over 5 days.

**Results:**

The lumpectomy technique as described herein varied by site and patient characteristics. The balloon applicator was implanted by the surgeon (91%) or a radiation oncologist (9%) during or up to 61 days post-lumpectomy (mean 22 days). A lateral approach was most commonly used (59%) for insertion of the applicator followed by an incision site approach in 27% of cases, a medial approach in 5%, and an inferior approach in 7%. A trocar was used during applicator insertion in 27% of cases. Local anesthetic, sedation, both or neither were administered in 45%, 2%, 41% and 11% of cases, respectively, during applicator placement. The prescription dose was delivered in 42 of 44 treated patients.

**Conclusions:**

Early stage breast cancer can be treated with breast conserving surgery and APBI using electronic brachytherapy. Treatment was well tolerated, and these early outcomes were similar to the early outcomes with iridium-based balloon brachytherapy.

## Background

The treatment of breast cancer has advanced considerably in the last two decades due to earlier detection, improved techniques for staging, development of alternative surgical approaches and radiation technologies, and coordination of multidisciplinary teams to implement multi-faceted treatment programs [1,2]. With the shift from mastectomy to breast-conserving surgery has come the reliance on post-operative adjuvant radiation therapy as an integral part of the local treatment regimen to the breast [3-5]. However, studies have shown that some patients opt for a mastectomy rather than lose time from family or work traveling to a distant radiation facility and/or undergoing a lengthy radiation treatment such as with conventional whole breast irradiation (WBI) [6-9]. The development of several techniques of accelerated partial breast irradiation (APBI) provides an alternative to WBI that reduces treatment time from weeks to days [6,10-12].

APBI studies using multiple interstitial catheters to deliver fractionated radiotherapy have demonstrated good long-term control rates and cosmesis with an acceptable safety profile at up to 12 years of follow up [12-14]. The use of a single balloon catheter for APBI has demonstrated good control rates, cosmesis and safety at up to 5 years followup [15-17] The majority of APBI techniques require the use of an ^192^Iridium source, which in turn requires a heavily-shielded radiation vault and a high dose rate (HDR) afterloader unit. These facilities are not present in many geographical areas of the United States due to the large capital expenditure [18]. An electronic X-ray source was developed as an alternative to the ^192^Iridium source for APBI. The electronic brachytherapy (EBT) system (Axxent^®^, Xoft, Inc., Sunnyvale, CA) uses a miniature HDR electronic 50 kV X-ray source for intracavitary APBI in a minimally shielded environment [18-20]. The electronic source mimics an ^192^Iridium source and provides an equivalent or higher dose rate with a steeper fall off of dose over distance [20].

In prospective studies of balloon-based APBI that enrolled patients prior to surgical implantation of the balloon applicator, approximately 30% of patients were ineligible for irradiation following implantation [20,21]. Nonconformance of the balloon to the lumpectomy cavity or inadequate margins between the balloon surface and the skin were the primary reasons for exclusion from brachytherapy treatment in both the ^192^Iridium and EBT studies. This report examines surgical techniques used during implantation of the EBT balloon applicator and contains patient data from the first multicenter EBT study. The initial publication of this study focused on treatment outcomes and characteristics of treated patients and tumors omitting critical surgical aspects of the study [20]. Herein we evaluate characteristics of both the ineligible and the eligible patients, the complete listing of adverse events and data from patient questionnaires. This report also provides an illustrated lumpectomy procedure that details optimal design of the lumpectomy cavity and overlying skin bridge in preparation for EBT balloon applicator placement.

## Methods

Overall results from the initial phase IV, prospective, multicenter, non-randomized EBT study were reported by Mehta, et al. [20], and the study methods were detailed in that publication. Data not presented in that paper regarding surgical details, characteristics of ineligible patients, adverse events, and patient questionnaires will be presented here, and the methods pertaining to those data are summarized below.

The study enrolled 65 patients at 10 study centers from March 2007 to March 2008. The Institutional Review Board at each of the 10 study sites approved the study protocol. The study was conducted in accordance with the Declaration of Helsinki and all applicable regulations. The patient selection criteria were based on the American Society of Breast Surgeons Consensus Statement for Accelerated Partial Breast Irradiation and the American Brachytherapy Society Breast Brachytherapy Task Group report [22,23]. Patients were initially screened for enrollment based on age (greater than 50 years), disease status (completely resected T1 invasive ductal cancer or ductal carcinoma in situ, less than 2 cm in diameter), availability for balloon applicator implantation within 5 weeks of their lumpectomy, and pathologically negative surgical margins on permanent section of at least 1 mm. Exclusion criteria included pregnancy, breast-feeding, a diagnosis of scleroderma, systemic sclerosis, active lupus, or a histological diagnosis of infiltrating lobular cancer. Each patient underwent informed consent prior to enrollment. After balloon applicator insertion, geometric conformance of the inflated balloon to the surgical cavity was verified using computerized tomography (CT) imaging as was a balloon surface to epidermal skin surface distance of at least 7 mm. Patients not meeting these criteria were excluded from treatment.

The EBT system was used to deliver intracavitary APBI to eligible patients. The EBT system uses an electronic, high dose rate, low energy (50 keV maximum energy) X-ray tube integrated into a flexible, multi-lumen catheter to deliver radiation. A sterile, disposable, single use balloon applicator functions as a guide for the X-ray source, and a mobile controller allows the X-ray source to be stepped within the balloon in order to tailor the radiation dose distribution to the tissue surrounding the balloon. A drainage system has been integrated into the Balloon Applicator to allow for suction of air or fluid from the lumpectomy cavity. The prescription dose delivered was 3.4 Gy twice daily for 5 days (10 fractions) to a distance of 1 cm beyond the balloon surface. Additional details about the system and treatment planning have been described in prior reports [18-20].

Patient and tumor characteristics were compared between groups of patients meeting all inclusion criteria and those ineligible for treatment. Factors affecting the success of implanting the balloon applicator and administering the prescribed radiation therapy were analyzed. Patients answered a questionnaire after implantation regarding their level of pain on a scale (normalized) from 0 (mild/none) to 6 (severe). Patients also answered a questionnaire post-treatment regarding their satisfaction with this radiation therapy on a scale from 0 (not satisfied) to 6 (very satisfied). Patient compliance with the APBI regimen and all study procedures was recorded. Adverse events were categorized by relationship to treatment and using the CTCAE 3.0 grading system [24]. Any recurrences and new cancers detected during the course of normal follow-up were recorded. Patients were evaluated at 1, 6 and 12 months and annually thereafter for up to 5 years.

## Results

In this phase IV study, 65 patients gave informed consent and were fully evaluated for eligibility to participate in the study. The inclusion and exclusion criteria were met by 44/65 (68%) patients, and 21 (32%) patients exited the study without treatment. Reasons for ineligibility included inadequate skin to balloon surface distance in 13 patients, balloon-to-cavity nonconformance in 3, age under 50 years in 1 patient, spontaneous balloon deflation in 2 patients (leading to withdrawal from the study), a positive axillary lymph node in 1 patient, and a positive margin on permanent pathologic analysis in 1 patient. Patient characteristics of the eligible and ineligible groups are shown in Table 1, and tumor characteristics of both groups are shown in Table 2. The majority of patients were post-menopausal Caucasian women with no prior history of cancer and no family history of breast cancer. The study was initially designed to follow patients for 6 months. Six months of follow-up data have been collected for 43/44 (98%) patients, and 1 patient was lost to follow-up after the 3-month visit. The protocol was amended to follow patients annually for up to 5 years, and 36/44 patients consented to the follow up phase of the study. One-year data are available on 36 patients, with a median duration of follow up of 394 days.

**Table 1:** Patient Demographics at Baseline

	Treated Patients	Ineligible Patients	P-Value
Number of Patients	44	21	--
Age: mean (range)	64 years (45-84)	64 Years (48-83)	p = NS
Ethnicity: n (%)			
Caucasian	38 (86.4%)	19 (90.5%)	p = NS
African-American	5 (11.4%)	2 (9.5%)	
Asian	1 (2.3%)	0 (0.0%)	
Menopausal Status: n (%)			
Pre-Menopausal	1 (2.3%)	1 (4.8%)	p = NS
Peri-Menopausal	2 (4.6%)	1 (4.8%)	
Post-Menopausal	41 (93.2%)	19 (90.5%)	
Prior history of cancer: n (%)			
Yes	8 (18.2%)	3 (14.3%)	p = NS
No	36 (81.8%)	17 (81.0%)	
Not reported	0 (0.0%)	1 (4.8%)	
Familial History of Breast Cancer: n (%)			
No Family History	28 (64%)	14 (66.7%)	p = NS
First Degree Relative With Breast Cancer	13 (30%)	6 (28.6%)	
Second Degree Relative With Breast Cancer	4 (9%)	0 (0%)	

NS = not significant

**Table 2:** Tumor Characteristics

	Treated (n = 44)	Ineligible (n = 21)	P-Value
Tumor Size: mean (range)	1.2 cm (0.2-2.8 cm)	1.2 cm (0.01-5.5 cm)	p = NS
Initial volume of excised tissue: mean (range)	63.6 cc (15-180 cc)	69.2 cc (35-144 cc)	p = NS
Excised volume after re-excision: mean (range)	32.6 cc (7-60 cc)	N/A	
Additional surgery to assure negative margins:			
Yes	9 (20.5%)	1 (4.8%)	p = NS
No	34 (77.3%)	20 (95.2%)	
Not reported or not applicable	1 (2.3%)	0 (0.0%)	
AJCC Class: n (%)			p = NS
Tis	12 (27.3%)	4 (19.1%)	
T1a	1 (2.3%)	3 (14.3%)	
T1b	8 (18.2%)	4 (19.1%)	
T1c	21 (47.7%)	8 (38.1%)	
T1mic	0 (0.0%)	1 (4.8%)	
T2	2 (4.6%)	0 (0.0%)	
Not reported	0 (0.0%)	1 (4.8%)	
Histopathologic Grade			p = NS
G1 Well Differentiated	12 (27.3%)	5 (23.8%)	
G2 Moderately Differentiated	18 (40.9%)	9 (42.9)%	
G3 Poorly Differentiated	10 (22.7%)	1 (4.8%)	
Grade Not Available	4 (9.1%)	5 (23.8%)	
Not reported	0 (0.0%)	1 (4.8%)	
Breast Cup Size			p = NS
B	12 (27.3%)	7 (33.3%)	
C	16 (36.4%)	4 (19.1%)	
D	11 (25.0%)	2 (9.5%)	
Not reported	5 (11.4%)	8 (38.1%)	
Lesion Location: Side			p = NS
Left Side	27 (61.4%)	14 (66.7%)	
Right Side	17 (38.6%)	7 (33.3%)	
Lesion Location: Vertical			p = NS
Upper	29 (65.9%)	14 (66.7%)	
Lower	10 (22.7%)	3 (14.3%)	
Midline	5 (11.4%)	4 (19.1%)	
Lesion Location: Horizontal			p = NS
Outer	26 (59.1%)	8 (38.1%)	
Inner	10 (22.7%)	6 (28.6%)	
Midline	8 (18.2%)	5 (23.8%)	
Not reported	0 (0.0%)	2 (9.5%)	

cc = cubic centimeters, cm = centimeters, NS = non-significant

The implantation procedure was successful, and the eligibility criteria were met in 44 patients with 3 exceptions: two patients with tumors of > 2.0 cm and one who was under the age of 50 were allowed treatment. A majority of balloon applicators were placed by a surgeon (91%) in a procedure room of the surgeon's office (48%), an outpatient clinic (30%), an operating room (11%) or another location (11%). A radiation oncologist placed the balloons in 9% of patients. A lateral approach was most commonly used (59%) for insertion of the applicator followed by an incision site approach in 27% of cases, a medial approach in 5%, and an inferior approach in 7%. A trocar was used during applicator insertion in 27% of cases. The procedure lasted a mean of 32 minutes (range 4-150 minutes) and was done on average 22 days (range 0-61 days) after the lumpectomy. Of the 44 patients who underwent balloon applicator placement, local anesthetic and sedation were administered in 18 patients at the time of applicator placement, local anesthetic without sedation in 20 patients, and sedation only in 1 patient. Five patients were not given any local anesthetic or sedation during balloon applicator placement.

The size and shape of each balloon applicator was predicated to best fit the cavity geometry of each individual patient in order to provide uniform contact between the wall of the applicator and the resultant surgical cavity. The 4-5 cm spherical balloon applicator was implanted in 84% of patients, the 3-4 cm spherical balloon in 2%, the 5-6 cm spherical balloon in 9% and the 5 × 7 cm ellipsoidal balloon in 5%. The mean volume of fluid instilled was 56.5 cc (range 35-110 cc depending on balloon size). After CT scan, the initial volumes were adjusted to optimize balloon conformance to the lumpectomy cavity. The final adjusted balloon volume was a mean of 57.7 cc (range 21-125 cc). Balloon conformance was inadequate in 3 patients leading to exclusion from the study. In two of these three patients, the physician attempted to place the balloon applicator, and in one patient the cavity was assessed by ultrasound and determined to not be adequate for placement of a balloon applicator. The time interval from lumpectomy to balloon applicator placement or ultrasound assessment in these 3 patients was 15, 19 and 16 days.

Assessment of the balloon and measurement of the distance from balloon surface to skin surface was evaluated at the time of implantation as well as prior to the first treatment. The distance was found to be inadequate in 13 patients leading to exclusion from treatment. Mean distance from balloon surface to skin surface was 25.4 mm (median 15.0 mm, range 8-96 mm) in the 44 patients who received treatment. For patients who reported CTC grade 1 and grade 2 skin toxicities, such as erythema, hypopigmentation, ecchymosis, and hyperpigmentation, the mean skin spacing assessed on CT prior to the first fraction was 14.8 mm (median 15.0 mm, range 6-28 mm).

The prescription dose was 34 Gy in 10 fractions over 5 days. The mean dwell times were 6.6, 7.8, 8.4, and 10.2 minutes in patients with a balloon size of 3-4 cm, 4-5 cm, 5-6 cm, and 5 × 7 cm, respectively [20].

Patients were asked to rate procedural pain on a scale of 1 (mild/none) to 6 (severe). In the 5 patients who did not receive local anesthetic or sedation during balloon applicator insertion, a mean score of 1.8 was tabulated. The mean score was 1.5 for the 20 patients who received local anesthesia, 0 for the patient administered sedation, and 2.1 for the 18 patients who received both local anesthesia and sedation. Patients were also asked to complete a survey about their participation in the study. Patient satisfaction was measured on a scale from 0 (not satisfied) to 6 (very satisfied). At one month post-treatment, the mean score for overall satisfaction with treatment was 5.8 (range 4-6). The mean score for overall satisfaction with study participation was 5.7 (range 2-6). The most common reason given by patients for participating in this study was physician recommendation (91% of patients) followed closely by a shortened radiation treatment time (86%) and delivery of radiation to a smaller area of the body (77%). Twelve of the patients (27%) indicated that having a local treatment facility was a factor in their decision to participate. Study centers were all located in or near major cities with cancer centers (Oklahoma City, OK, Evergreen Park, IL, Chicago, IL, Seattle, WA, Providence, RI, San Mateo, CA, Marietta, GA, New York City, NY, White Plains, NY, Silver Spring, MD).

Adverse events were generally mild and manageable during treatment and over a median duration of follow up of 394 days. Table 3 reviews Grade 2-3 adverse events as reported by Mehta, et al. [20], and provides all Grade 1 adverse events. There were no serious adverse events. Four patients had CTC grade 3 toxicities (blistering in 1, breast tenderness in 1, and moist desquamation in 2) with subsequent resolution in the post-treatment period as described in detail elsewhere [20,25]. All other adverse events were Grade 1 or 2.

**Table 3:** Adverse Events

Adverse Event	Grade 1	Grade 2	Grade 3
Blistering	2 (4.5%)	0	1 (2.3%)
Bruising	1 (2.3%)	0	0
Desquamation, Dry	1 (2.3%)	1 (2.3%)	0
Desquamation, Moist	1 (2.3%)	0	2 (4.5%)
Drainage, Serosanguinous	1 (2.3%)	0	0
Dry Skin (Breast)	2 (4.5%)	0	0
Ecchymosis	1 (2.3%)	0	0
Erythema, redness/rash	19 (43.2%)	8 (18.2%)	0
Fatigue	2 (4.5%)	4 (9.1%)	0
Fibrosis	1 (2.3%)	1 (2.3%)	0
Firmness (Breast tissue)	2 (4.5%)	0	0
Firmness (Skin)	2 (4.5%)	0	0
Hyperpigmentation / Hypopigmentation / Skin Discoloration	6 (13.6%)	3 (6.8%)	0
Induration	3 (6.8%)	0	0
Infection	0	2 (4.5%)	0
Itching / Pruritis	4 (9.1%)	0	0
Mass, 2.5 cm, non-calcified	1 (2.3%)	0	0
Pain (Rib)	1 (2.3%)	0	0
Pain / Tenderness / Discomfort	7 (15.9%)	5 (11.4%)	1 (2.3%)
Seroma	0	2 (4.5%)	0
Swelling	2 (4.5%)	0	0
Wound complication, non-infection	1 (2.3%)	0	0

Number (%) of treated patients reporting each adverse event by CTC grade (N = 44)

CTC = Common Terminology Criteria, cm = centimeter

## Discussion

The American Society of Breast Surgeons and the American Brachytherapy Society have published guidelines for the screening and selection of patients for APBI [22,23], and these guidelines formed the basis for patient selection in the EBT multicenter study [20]. Of the 65 patients that met the initial screening requirements, 44 patients met all eligibility criteria and were treated. The majority of the 21 patients not eligible for treatment were disqualified at the time of implantation for inadequate balloon conformance to the tumor cavity or inadequate distance from skin to balloon surface. In this study, patients were enrolled and screened post-lumpectomy. For patients undergoing lumpectomy with the intention of pursuing APBI, a surgeon should be able to determine at the time of lumpectomy whether a patient is likely to meet the eligibility requirements for successful post-operative balloon implantation [11]. The surgical technique used at the time of lumpectomy can help promote successful balloon spacing and help the patient meet the eligibility criteria. Careful attention to the depth of the lesion from the skin using ultrasound measurements is needed for optimal design of the lumpectomy and the post-lumpectomy cavity, which will determine balloon position. Many patients have tumors too close to the skin or more extensive than appreciated on pre-op and intra-op imaging. These patients end up with a narrow skin bridge or positive margins and would not be candidates for APBI. With rather simple modifications of certain oncoplastic techniques, the overlying skin can be excised or margin width increased in such a way that the deeper 270 degrees of the lumpectomy base is still ideally radiated and treated optimally with APBI. This requires joint pre-op planning by the surgical and radiation teams. This methodology increases dramatically the number of acceptable APBI candidates and decreases poor balloon placement and conformity issues.

**Figure 1:**
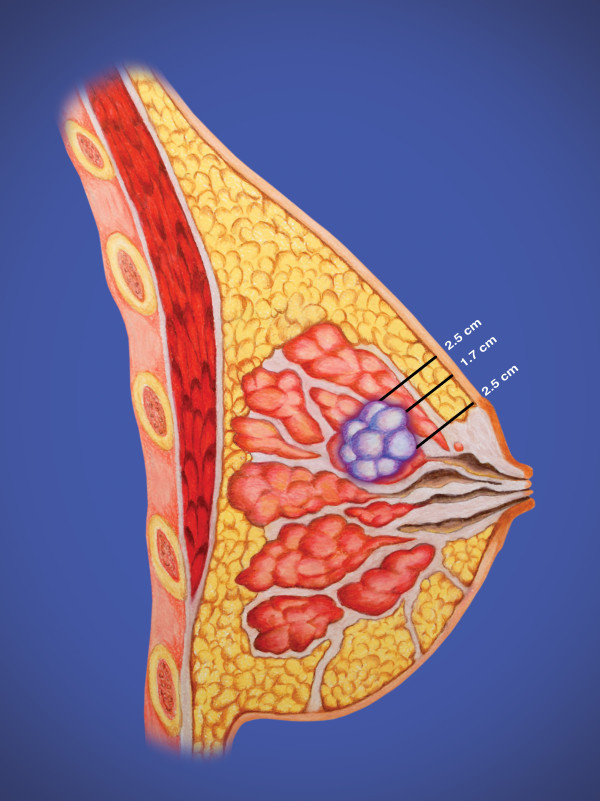
**Side view of a small breast cancer just superior to the areolar edge**.

**Figure 2:**
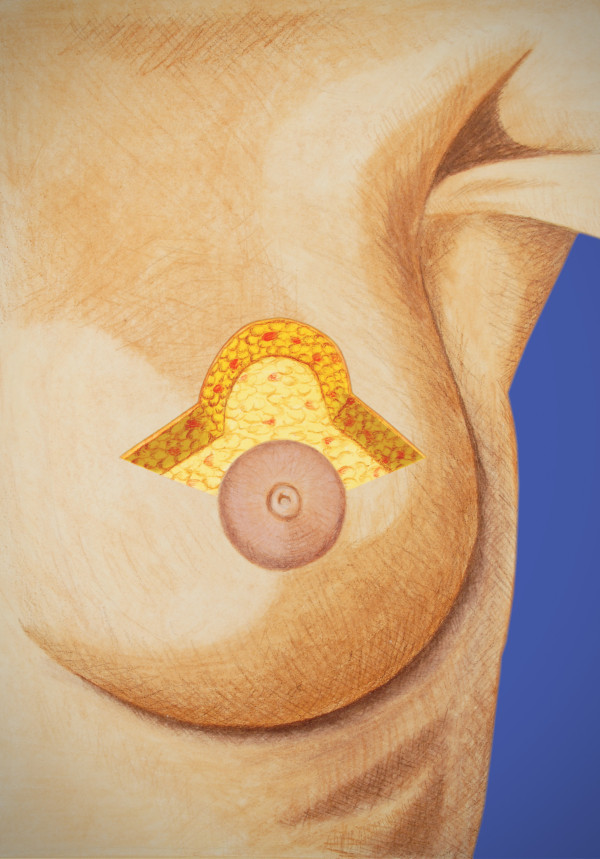
**Modified bat wing approach**. This approach allows excision of the tumor and a thin skin bridge but preserves posterior breast tissue for lung spacing.

An illustrated lumpectomy procedure that details optimal design of the lumpectomy cavity and overlying skin bridge in preparation for EBT balloon applicator placement was developed at this site during this trial and is included as an example; these procedures may need to be modified given individual differences between patients. Figure 1 illustrates a lesion just superior to the areola. Since a standard lumpectomy would remove approximately 1 cm of normal breast surrounding the lesion, the overlying skin bridge would be too thin for balloon-based APBI. The EBT balloon appears to be slightly thicker than other APBI balloons. When the balloon is inflated, the tissue bridge superficial to the balloon tends to compress to a greater degree with the Axxent balloon than with other APBI balloons. Consequently the overlying skin bridge should be a minimum of 1.2-1.5 cm. In this example it is necessary to excise all breast and skin within an area of less than 2.5 cm of the skin surface. As seen in Figure 2, this can readily be accomplished by using modifications of the standard oncoplastics incisions. Since the lesion in this case was close to the areolar edge, we chose a bat wing mastopexy. With this approach for APBI, we are also interested in the tissue depth between the back side of the balloon and the underlying ribs and lung. By not carrying the excision to the full thickness commonly illustrated in oncoplastics descriptions [26], we preserve some breast tissue to add to the posterior spacing and offer more lung protection.

Retractors were not used in order to avoid beveling toward the skin. Instead of retractors, two prolene stitches were placed lateral and medial to the lesion, and, as these were pulled upward, electrocautery was used to cut toward the lesion. The flat superficial surface is where the skin island is located. Beveling outward is minimal, and the bottom side of the removed tissue "V"s downward, resulting in a shape similar to that of a typical solitaire cut diamond. It is important to have supporting breast tissue structure, especially in older women.

**Figure 3:**
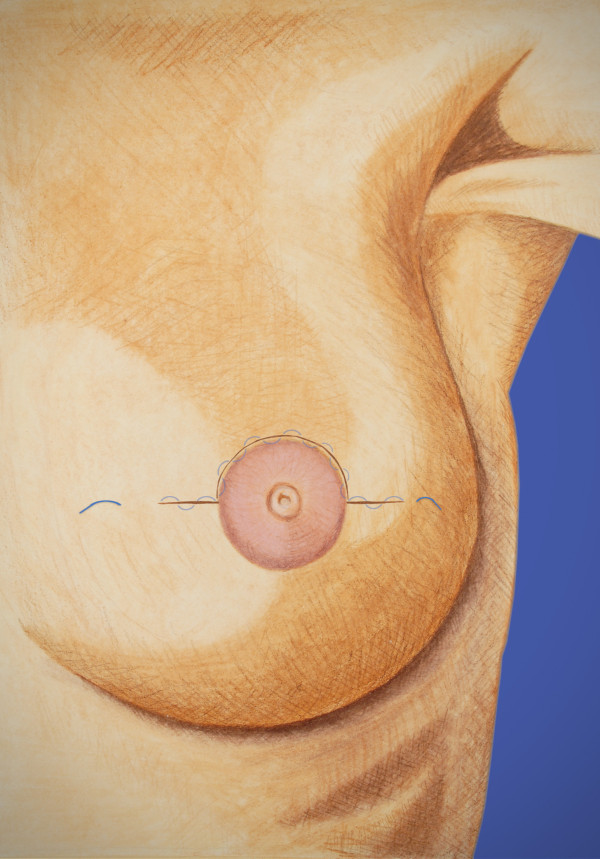
**Closed incision with several layers of running barbed suture**.

**Figure 4:**
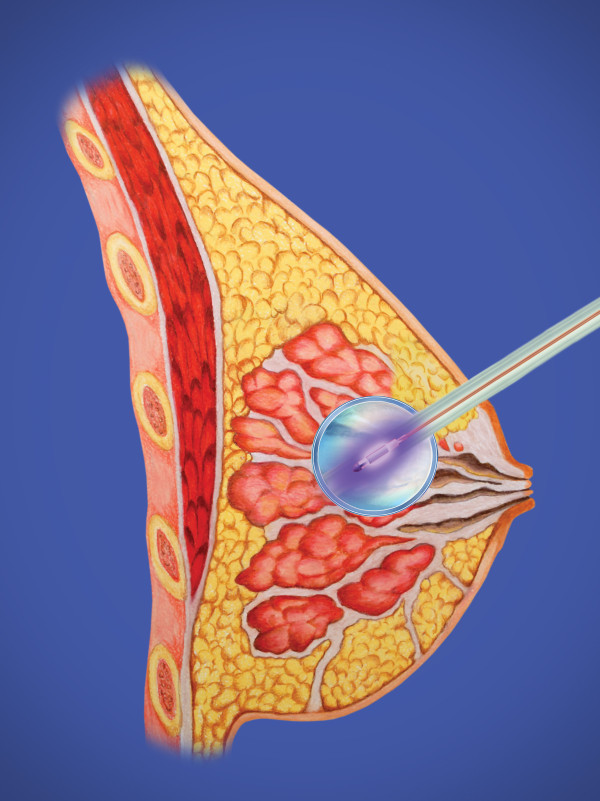
**Electronic brachytherapy balloon inflated with source active in place with adequate skin spacing**.

Optimal closures (Figure 3) were performed in three layers and began deeper than conventional closure, at least 12-15 mm from the skin, making sure to take a generous thickness of tissue. The dense superficial fascia of the breast and superficial dense breast tissue when present will hold the sutures for this layer most effectively. An option for a resilient closure, which will not collapse during balloon inflation, is the use of a running barbed suture, such as a Quill suture, for each of these layers. An inflated balloon exerts pressure against the skin, and an adequate skin bridge will maintain the distance from the balloon to the skin surface. The greater the volume of the balloon, with the attendant compression of adjacent breast tissue, the more fully the target breast tissue will be irradiated to achieve margin sterilization. These techniques usually place the center of the inflated balloon slightly deeper than the original tumor center but maintain all residual breast tissues within 1 cm of the lumpectomy tightly compressed to the balloon surface for optimal therapy (Figure 4). In some patients, implantation of the balloon may be possible at the time of lumpectomy. Alternatively some surgeons have used a removable placeholder device, a cavity evaluation device (CED), to preserve the cavity while fashioning an easily accessible tract between the cavity and the skin surface [11]. This can facilitate balloon implantation in the post-operative period. Post-operative antibiotic coverage is used in this circumstance to lessen the risk of infection. As with other devices, the surgical technique should be be discussed with the radiation oncologist to enable proper treatment planning.

In this study, balloon applicators were implanted up to 61 days post-lumpectomy. In the 3 patients with inadequate balloon conformance to cavity geometry, the time from lumpectomy to implantation for each was 15, 16 and 19 days, which was less than the mean for the study group. Tumor characteristics were similar between the eligible group and ineligible group. The age range of patients was 45-84 years, with a range of lesion sizes from 0.2-2.8 cm. Breast cup sizes were evenly distributed between B, C, and D. No apparent trends were noted between eligible and ineligible patients although the sample size limits statistical analysis.

The prescription dose of 34 Gy was delivered in 42/44 patients, and 2/44 patients received total doses of 30.6 and 33.96 Gy [20]. Treatment was well tolerated, and adverse events were similar to adverse events with other forms of APBI. Complications associated with the implantation of the balloon applicator are similar to complications reported during the insertion of a post surgical drain [27]. This type of complication has also been reported with ^192^Ir-based balloon brachytherapy [11,15,16]. During this EBT study one patient had incisional redness/drainage at 3 months post-treatment, 2 patients had infection at 3 and 6 months, respectively, and 2 patients had seromas at or within 4 weeks of treatment. The patients who were diagnosed with infection were treated with oral antibiotics, and the infections resolved. Study sites used standard wound care procedures at the skin exit site, which included the use of topical antibiotic ointment and/or hydrogen peroxide. Patients were instructed to wear a surgical bra and avoid showering during the 5-day treatment period. Patient compliance with the treatment regimen was excellent. Patients expressed satisfaction with the conduct of the study as well as the delivery of the radiotherapy based on a questionnaire given at follow-up visits. Mehta, et al., reported cosmesis to have been evaluated as good to excellent by 80% of patients at 6 months [20].

Our initial experience with both a new balloon applicator and a novel radiation source parallels the initial experience with the ^192^Ir-based balloon brachytherapy [17,21], and the two studies are similar in enrollment, subsequent treatment eligibility, patient characteristics, tumor characteristics, and cosmetic results following EBT and ^192^Ir-based brachytherapy as summarized in Table 4[17,20,21]. The range and frequency of adverse events were similar for erythema, pain, and infection. However, catheter site drainage was reported in 52% of patients following ^192^Ir-based brachytherapy but was not reported with EBT. This may be due to differences in the design of the balloon applicators. The most significant difference with the EBT system is the use of an electronic high dose rate, low energy X-ray source to generate radiation, which eliminates the issues involved in the handling and storage of radioactive isotopes [18]. Many radiation treatment centers as well as community hospitals across the United States lack the funds and infrastructure to maintain isotopes or build heavily shielded treatment rooms that are required for the delivery of HDR brachytherapy. The EBT system does not require a heavily shielded treatment room or an HDR brachytherapy afterloader. The EBT system should provide a means that will allow women not currently able to travel to radiation facilities with HDR brachytherapy afterloaders to be treated within their local communities and receive state of the art breast radiotherapy as an adjunct to modern breast conserving surgery.

**Table 4:** Comparison electronic brachytherapy (EBT) and ^192^Iridium brachytherapy (IBT)

Patient/Tumor Characteristics	EBT	IBT
Number of Patients Enrolled	65	75
Number of Patients Treated	44 (67.7%)	43 (57.3%)
Age: mean (range)	64 years (45-84)	69 years (50-90)
Menopausal Status: n (%)		
Pre-Menopausal	1 (2.3%)	0
Peri-Menopausal	2 (4.6%)	2 (5%)
Post-Menopausal	41 (93.2%)	41 (95%)
Tumor Size: mean (range)	1.2 cm (0.2-2.8 cm)	1.0 cm
AJCC Class: n (%)		
Tis	12 (27.3%)	0
T1a	1 (2.3%)	9 (21%)
T1b	8 (18.2%)	16 (37%)
T1c	21 (47.7%)	18 (42%)
T2	2 (4.6%)	0
Histopathologic Grade		
G1 Well Differentiated	12 (27.3%)	17 (40%)
G2 Moderately Differentiated	18 (40.9%)	16 (37%)
G3 Poorly Differentiated	10 (22.7%)	6 (14%)
Grade Not Available	4 (9.1%)	4 (9%)

Minimum Distance from Balloon to Skin Surface	6 mm	5 mm

Breast Cup Size		
A	0	1 (2%)
B	12 (27.3%)	9 (21%)
C	16 (36.4%)	15 (35%)
D+	11 (25.0%)	11 (26%)
Not reported	5 (11.4%)	7 (16%)

**Cosmesis**	**EBT**	**IBT**
Good - Excellent cosmesis at 1 month	35/44 (80%)	38/43 (88%)
Good - Excellent cosmesis at 1 year	24/32 (75%)	--
Good - Excellent cosmesis at 5 years	--	35/43 (81%)

**Adverse Events in > 2 patients**	**EBT ^a^**	**IBT ^b^**
Blistering	3 (6.8%)	2 (3.7%)
Bruising / hematoma	1 (2.3%)	3 (5.6%)
Catheter Site Drainage	0	28 (51.9%)
Desquamation, Dry	2 (4.5%)	7 (13.0%)
Desquamation, Moist	3 (6.8%)	3 (5.6%)
Dry Skin (Breast)	2 (4.5%)	6 (11.1%)
Ecchymosis	1 (2.3%)	17 (31.5%)
Edema (breast)	0	8 (14.8%)
Erythema, redness/rash	27 (61.4%)	31 (57.4%)
Fibrosis	2 (4.5%)	3 (5.6%)
Hyperpigmentation / Hypopigmentation	9 (20.5%)	5 (9.3%)
Induration	3 (6.8%)	1 (1.9%)
Infection	2 (4.5%)	2 (3.7%)
Itching / Pruritis	4 (9.1%)	5 (9.3%)
Pain, tenderness, discomfort	13 (29.5%)	23 (42.6%)
Rash	0	4 (7.4%)
Seroma	2 (4.5%)	6 (11.1%)
Skin Irritation	0	3 (5.6%)

Patient and tumor characteristics at baseline, cosmesis at 1 month and 1 or 5 years, and adverse events (breast and skin symptoms and signs) following EBT at 6 months as reported herein and by Mehta, et al., ^20 ^or IBT at 1 month as reported by Keisch, et al. ^21^

^a^Adverse events in 1 or 2 patients in the EBT study (not listed above) included firmness of breast tissue, skin firmness, non-calcified mass (2.5 cm), rib pain, serosanguinous drainage, swelling, and wound complication (non-infection).

^b^Adverse events in 1 or 2 patients in the IBT study (not listed above) included abscess, eschar, mastitis, serosanguinous drainage, telangiectasia, and vasodilatation.^24^

## Conclusions

Early stage breast cancer can be treated with breast conserving therapy and accelerated partial breast irradiation using electronic brachytherapy. Treatment was well tolerated, and these early outcomes were similar to the early outcomes with iridium-based balloon brachytherapy.

## Abbreviations

APBI: accelerated partial breast irradiation; CED: cavity evaluation device; CT: computerized tomography; CTC: Common Terminology Criteria; cm: centimeter; EBT: electronic brachytherapy; Gy: gray; HDR: High dose rate; Inc: incorporated; Ir: Iridium.

## Competing interests

Darius Francescatti is a paid consulting surgical medical director for Xoft Inc. All other authors declare that they have no competing interests.

## Authors' contributions

All authors contributed to treatment of patients, collection of data, review of results and manuscript, and approval of the final draft.
